# Editorial: Reviews in neglected tropical infectious diseases

**DOI:** 10.3389/fmicb.2023.1196838

**Published:** 2023-04-26

**Authors:** Suvankar Ghorai

**Affiliations:** Department of Microbiology, Raiganj University, Raiganj, WB, India

**Keywords:** NTD, Leprosy, Dengue, Chikungunya, Schistosomiasis, Lymphatic Filariasis

Neglected Tropical Diseases (NTDs) are the varying tropical infections that are prevalent in the low-income and developing countries of Asia, Africa, and America (Hotez et al., 2020). A wide variety of pathogens like parasitic worms, bacteria, viruses, and protozoans are responsible for causing NTDs. Some NTDs are asymptomatic, and some have longer periods of incubation; hence, they remain neglected and unrealized. NTDs take a tremendous toll on human health as they can create immense pain, disability, and even death.

WHO has estimated that yearly more than a billion people are affected by this disease in ~150 countries where this disease is endemic. Populations affected by NTDs face problems such as poverty, inadequate sanitation, and living in close proximity to infectious vectors and livestock. It is also associated with social stigma; this makes the treatment process more complex.

Approximately 20 diseases have been recognized as NTDs which include Buruli ulcer, Chagas disease, Dengue and Chikungunya, Dracunculiasis (Guinea-worm disease), Echinococcosis, foodborne trematodiases, human African trypanosomiasis (sleeping sickness), Leishmaniasis, Leprosy (Hansen's disease), Lymphatic Filariasis, Mycetoma, Chromoblastomycosis and other deep mycoses, Onchocerciasis (river blindness), Rabies, Scabies and other ectoparasitoses, Schistosomiasis, soil-transmitted helminthiases, snakebite envenoming, taeniasis/cysticercosis, Trachoma, and Yaws and other endemic treponematoses (Feasey et al., 2010). Diseases like Dracunculiasis, Lymphatic Filariasis, Onchocerciasis, Schistosomiasis, Leprosy, Visceral leishmaniasis (kala-azar), and Yaws have been effectively targeted for control and eradication through mass administration of proper medication and intervention by controlling the vectors (World Health Organization, 2010).

Neglected zoonotic diseases (NZDs) are a subset of NTDs that are transmitted among humans and other vertebrates directly or indirectly via vectors like mosquitoes spreading malaria, Dengue, etc. (Mackey et al., 2014). In sub-Saharan countries, more than one-third of pregnant women are estimated to be infected with an NTD agent, and it can severely impact maternal and child health if not detected and treated appropriately. This includes aggravated blood loss during childbirth, which can be fatal, mother-to-child disease transmission, and gender-specific consequences of NTDs such as female urogenital Schistosomiasis.

NTDs can be eradicated by throwing proper light on this issue, which includes education, sanitation, hygiene, research for vaccines and drugs, and better diagnosis (Bodimeade et al., 2019).

Looking at the emergence of these diseases, there is a need to rethink and relook carefully to examine the present scenario of these diseases so that proper emphasis can be given, and appropriate measures can be decided.

The editor of this present topic would like to acknowledge all the authors for their valuable contributions in the form of manuscripts to address different aspects of neglected diseases.

Kalkal and Das have given a significant approach toward the highly prevalent disease malaria which is still neglected. Malaria is a febrile illness caused by infected female Anopheles mosquitoes and its causative parasite is *Plasmodium* species; it is prevalent in tropical and subtropical regions. WHO reported 247 million cases of malaria globally in 2021 (World Health Organization, 2021). Although Malaria is preventable by vector control, chemo-preventive therapies, chemoprophylaxis, and vaccination, still every year huge numbers of people are infected and lose their life. RTS,S/AS01, commonly known as Mosquirixis, is the only vaccine known to reduce the malarial burden in children living in high Malaria transmission areas (Laurens, 2020). It is active against the *Plasmodium falciparum* parasite, the deadliest Malaria parasite globally and the most prevalent in Africa. In America, South-East Asia, and the Eastern Mediterranean zone, *Plasmodium vivax* causes most of the cases. In the year 2020, the Sub-Saharan region faced half of global malarial death. The role of B-cells in creating an immune response in *Plasmodium* infection is crucial. The antibodies produced during malarial infection have a very short life span. More research is required on the novel B-cell subtype (regulatory B-cells), which can regulate the immune response in malarial infection. The long-lived antibodies produced by B-cells in the germinal center can pave a path for Malarial vaccine development. The potency in targeting the B-cell is a crucial area of study in serious and life-threatening Malaria cases (Wipasa et al., 2010).

Kumari and Sinha have focused on the importance of culture-based research in the field of *Plasmodium vivax* cultural techniques, which can help in the understanding of host-parasite interaction. The knowledge of the pathogenesis of *P. vivax* is a setback because of the absence of its vigorous and continuous culture. The pre-existing cultural techniques for *P. vivax* parasites include a hassle in persistence in culture and understanding parasite biology that demands efficient culture conditions for *P. vivax* studies, which will provide a better understanding of the host-parasite interaction (Roobsoong et al., 2015). In order to achieve this, various factors like host-parasite interaction and environmental conditions like effected reticulocytes, osmotic stability after parasite invasion, and abundant iron in the reticulocytes need to be studied.

Knight et al. have shed light on Leishmaniasis which is a fatal and ignored disease. Leishmaniasis is the third most cardinal NTD and is prevalent in low-income countries (Molyneux et al., 2017). This disease is caused due to *Leishmania* and is spread by the bites of sandflies. More than 12 million people are infected with Leishmania spp.; additionally, 350 million are at risk of infection (Torres-Guerrero et al., 2017). The symptoms of this disease include large, slow-healing ulcers, fever, weight loss, and abdominal swelling. The diagnosis of Leishmaniasis is a major challenge as its symptoms are similar to other diseases like tuberculosis, malaria, typhoid, etc., and there is a wide spectrum of clinical manifestations and co-infection with other diseases. The burden of Leishmaniasis is high in some Middle Eastern countries, but in countries like Saudi Arabia, the rate of infection has diminished in this decade. The conventional treatment for Leishmania parasite includes medicines like Amphotericin, Miltefosine, Pentavalent antimonials such as sodium stibogluconate, Paromomycin for cutaneous leishmaniasis treatment, thermotherapy, cryotherapy, and laser therapy (Garza-Tovar et al., 2020). These conventional medications also have negative impacts on patients' health including nephrotoxicity and resultant kidney failure, leading to broad research in the field of liposomes, nanoparticles, and emulsions to provide better medication alternatives. Researchers are also using the CRISPR approach to attenuate the parasite to provide long-term and safer immunization as compared to the pre-existing leishmanization procedure. The need for the development of a pan-Leishmania vaccine is essential in spite of its epidemic geographical location. Many researchers are also trying to develop alternative therapies using animal toxins, insecticide nets, immuno-therapy, etc.

Gaspar-Castillo et al. have described the necessity of the development of advanced diagnosis for Zika and Dengue. Dengue is currently endemic in more than 100 countries including Africa, America, and South-East Asia. Dengue and Zika virus can be transmitted to humans through mosquito (*Aedes aegypti*) bites; however, evidence of maternal transmission has also been found. DENV and ZIKV flavivirus showed high structural similarity and cross-reactive immune response, which results in false positives during serological tests, precisely in secondary infections. The present serological test includes NS1 and E proteins as antigenic targets (Tan et al., 2019). The precision and accuracy of the test depend on the infection stage. The structure and immunology-based E and NS1 protein epitopes are well-studied as they are targets for neutralizing antibody response; still, advanced research is needed to understand novel non-conserved linear epitopes in E (i.e., E DIII) and NS1 proteins which can enhance the specificity of serological testing regardless of the technological platform. There is a necessity for the justified seroprevalence estimation for widespread vaccination against Dengue and Zika.

## Author contributions

SG conceptualized the content and wrote the manuscript.

## References

[B1] Bodimeade C. Marks M. Mabey D. (2019). Neglected tropical diseases: elimination and eradication. Clin. Med. 19, 157–160. 10.7861/clinmedicine.19-2-157 30872302 PMC6454364

[B2] Feasey N. Wansbrough-Jones M. Mabey D. C. Solomon A. W. (2010). Neglected tropical diseases. Br. Med. Bull. 93, 179–200. 10.1093/bmb/ldp046 20007668

[B3] Garza-Tovar T. F. Sacriste-Hernández M. I. Juárez-Durán E. R. Arenas R. (2020). An overview of the treatment of cutaneous leishmaniasis. Faculty Rev. 9, 28. 10.12703/r/9-28 33659960 PMC7886081

[B4] Hotez P. J. Aksoy S. Brindley P. J. Kamhawi S. (2020). What constitutes a neglected tropical disease? PLoS Negl. Trop. Dis. 14, e0008001. 10.1371/journal.pntd.0008001 31999732 PMC6991948

[B5] Laurens M. B. (2020). RTS,S/AS01 vaccine (Mosquirix™): an overview. Hum. Vacc. Immunother. 16, 480–489. 10.1080/21645515.2019.1669415 31545128 PMC7227679

[B6] Mackey T. K. Liang B. A. Cuomo R. Hafen R. Brouwer K. C. Lee D. E. (2014). Emerging and reemerging neglected tropical diseases: a review of key characteristics, risk factors, and the policy and innovation environment. Clin. Microbiol. Rev. 27, 949–979. 10.1128/CMR.00045-14 25278579 PMC4187634

[B7] Molyneux D. H. Savioli L. Engels D. (2017). Neglected tropical diseases: progress towards addressing the chronic pandemic. Lancet 389, 312–325. 10.1016/S0140-6736(16)30171-4 27639954

[B8] Roobsoong W. Tharinjaroen C. S. Rachaphaew N. Chobson P. Schofield L. Cui L.. (2015). Improvement of culture conditions for long-term *in vitro* culture of *Plasmodium vivax*. Malaria J. 14, 297. 10.1186/s12936-015-0815-z 26243280 PMC4524445

[B9] Tan L. K. Wong W. Y. Yang H. T. Huber R. G. Bond P. J. Ng L. C.. (2019). Flavivirus cross-reactivity to dengue nonstructural protein 1 antigen detection assays. Diagnostics 10, 11. 10.3390/diagnostics10010011 31878299 PMC7167843

[B10] Torres-Guerrero E. Quintanilla-Cedillo M. R. Ruiz-Esmenjaud J. Arenas R. (2017). Leishmaniasis: a review. F1000 Res. 6, 750. 10.12688/f1000research.11120.1 28649370 PMC5464238

[B11] Wipasa J. Suphavilai C. Okell L. C. Cook J. Corran P. H. Thaikla K.. (2010). Long-lived antibody and B cell memory responses to the human malaria parasites, *Plasmodium falciparum* and *Plasmodium vivax*. PLoS Pathogens 6, e1000770. 10.1371/journal.ppat.1000770 20174609 PMC2824751

[B12] World Health Organization (2010). First WHO Report on Neglected Tropical Diseases: Working to Overcome the Global Impact of Neglected Tropical Diseases. Geneva: WHO.

[B13] World Health Organization (2021). World Malaria Report 2021. Available online at: https://www.who.int/teams/global-malaria-programme/reports/world-malaria-report-2021 (accessed March 15, 2023).

